# GABA-A receptor differences in schizophrenia: a positron emission tomography study using [^11^C]Ro154513

**DOI:** 10.1038/s41380-020-0711-y

**Published:** 2020-04-15

**Authors:** Tiago Reis Marques, Abhishekh H. Ashok, Ilinca Angelescu, Faith Borgan, Jim Myers, Anne Lingford-Hughes, David J. Nutt, Mattia Veronese, Federico E. Turkheimer, Oliver D. Howes

**Affiliations:** 1 Psychiatric Imaging Group, MRC London Institute of Medical Sciences (LMS), Imperial College London, London, UK; 2 Department of Psychosis Studies, Institute of Psychiatry, Psychology and Neuroscience, King’s College London, London, UK; 3 Faculty of Medicine, Imperial College London, London, UK; 4 Neuropsychopharmacology Unit, Centre for Psychiatry, Division of Brain Sciences, Imperial College London, London, UK; 5 Department of Neuroimaging, Institute of Psychiatry, Psychology and Neuroscience, King’s College London, London, UK

**Keywords:** Schizophrenia, Neuroscience

## Abstract

A loss of GABA signaling is a prevailing hypothesis for the pathogenesis of schizophrenia. Preclinical studies indicate that blockade of the α5 subtype of the GABA receptor (α5-GABA_A_Rs) leads to behavioral phenotypes associated with schizophrenia, and postmortem evidence indicates lower hippocampal α5-GABA_A_Rs protein and mRNA levels in schizophrenia. However, it is unclear if α5-GABA_A_Rs are altered in vivo or related to symptoms. We investigated α5-GABA_A_Rs availability in antipsychotic-free schizophrenia patients and antipsychotic-medicated schizophrenia patients using [^11^C]Ro15-4513 PET imaging in a cross-sectional, case–control study design. Thirty-one schizophrenia patients (*n* = 10 antipsychotic free) and twenty-nine matched healthy controls underwent a [^11^C]Ro15-4513 PET scan and MRI. The α5 subtype GABA-A receptor availability was indexed using [^11^C]Ro15-4513 PET imaging. Dynamic PET data were analyzed using the two-tissue compartment model with an arterial plasma input function and total volume of distribution (*V*_T_) as the outcome measure. Symptom severity was assessed using the PANSS scale. There was significantly lower [11C]Ro15-4513 *V*_T_ in the hippocampus of antipsychotic-free patients, but not in medicated patients (*p* = 0.64), relative to healthy controls (*p* < 0.05; effect size = 1.4). There was also a significant positive correlation between [^11^C]Ro15-4513 *V*_T_ and total PANSS score in antipsychotic-free patients (*r* = 0.72; *p* = 0.044). The results suggest that antipsychotic-free patients with schizophrenia have lower α5-GABAARs levels in the hippocampus, consistent with the hypothesis that GABA hypofunction underlies the pathophysiology of the disorder.

## Introduction

A leading hypothesis proposes that reduced gamma-aminobutyric acid (GABA) signaling in frontotemporal brain regions underlies the pathogenesis of schizophrenia [1–3]. This hypothesis is supported by the postmortem literature showing that schizophrenia patients relative to controls show lower mRNA and protein levels of the synthetic enzyme GAD67 [4], lower levels of the GABA membrane transporter (GAT1) [5], and lower expression and cell density of GABAergic interneurons relative to controls [3, 6]. In vivo studies have reported a reduction in concentrations of GABA in cerebrospinal fluid concentrations in first-episode psychotic patients [7]. By contrast proton magnetic resonance spectroscopy (1H-MRS) have yielded discrepant findings, potentially due to the technical difficulties in accurately resolving GABA on MRS spectra and its complex relationship with synaptic GABA [8, 9]. GABA acts on two different subtypes of receptors, GABA-A and GABA-B. The GABA-A receptor is responsible for the majority of the physiological actions of GABA and it includes receptor subtypes made of different combinations of α, β, γ, δ, ε, r, π, and θ subunits, organized in a pentameric structure arranged around a central pore [10].

The diversity in subunit composition and region-specific distribution allows GABA-A receptors to have distinct functional and physiological roles [11]. Studies using [^11^C]flumazenil and [^123^I]iomazenil to index GABA receptor levels have not found differences between schizophrenia patients and healthy controls [12–15]. However, these radiotracers are not GABA subtype selective and show similar levels of uptake both in neocortex and limbic regions, which means they may be unable to detect differences in specific GABA receptor subtypes [16, 17]. In contrast, the inverse GABA-A receptor agonist [^11^C]Ro15-4513 is a radioligand that binds with ten times higher affinity (~0.5 nM) to the α5 subunit of the GABA receptor (α5-GABA_A_Rs) when compared with other subunits (~10 nM) [16, 18, 19]. Although α5-GABA_A_Rs represents <5% of all GABA-A receptors [20], they are particularly concentrated in limbic regions [21]. In the hippocampus, a key brain region implicated in the pathogenesis of schizophrenia [22], 25% of GABA-A receptors are α5-GABA_A_Rs [21]. Importantly, changes in α5-GABA_A_Rs have been reported in schizophrenia patients, with postmortem studies showing lower mRNA and protein levels of this receptor [23, 24]. Moreover, an α5-GABA_A_Rs positive allosteric modulator (PAM) has been shown to reverse dopaminergic hyperactivation and behavioral sensitivity to psychostimulants in methylazoxymethanol (MAM)-treated animals, a preclinical model that reproduces many phenotypes seen in schizophrenia [25]. In contrast, the blockade of the α5-GABA_A_Rs impairs latent inhibition [26] and reduces prepulse inhibition to startle [27], behavioral phenotypes associated with schizophrenia. Whilst these converging lines of evidence implicate the α5-GABA_A_Rs in schizophrenia, the only previous PET study using [^11^C]Ro15-4513 to assess α5-GABA_A_Rs availability showed no differences in binding potential (BP) between patients with schizophrenia and controls [28]. However, this study used a brain reference region approach, which is associated with an underestimation of BPs, and the potential confound of systematic differences in GABA availability in the reference region as well [29]. Therefore, it remains unclear if α5-GABA_A_Rs availability is altered in schizophrenia patients in vivo when controlling for these methodological issues.

In view of this, we sought to investigate α5-GABA_A_Rs availability in patients with schizophrenia using [^11^C]Ro15-4513 PET imaging with an arterial input function. We hypothesized that both medicated and antipsychotic-free schizophrenia patients would have lower GABA-A receptor availability in the hippocampus when compared with healthy controls, and that GABA-A receptor availability would be associated with symptom severity.

## Material and methods

### Participants

The study protocol was approved by the local Research Ethics Committee and the Administration of Radioactive Substances Advisory Committee, United Kingdom. All participants gave written informed consent prior to study participation. To control for potential sex differences in α5-GABA_A_Rs availability [30] we specifically investigated male volunteers. Since antipsychotic medication may influence GABA function [31, 32] we recruited two groups of patients, one taking antipsychotic treatment and the other antipsychotic free, and compared each group with matched healthy controls. Patients on antipsychotics were on a stable dose of antipsychotics for at least 4 weeks prior to the scan day. A total of 25 medicated patients with schizophrenia or schizoaffective disorder and 19 age-matched healthy controls were recruited. A further group of ten antipsychotic-free patients with schizophrenia or schizoaffective disorder and ten age-matched healthy controls were also recruited. All patients had capacity to give informed written consent and satisfied DSM-V diagnostic criteria for schizophrenia or schizoaffective disorder as determined by the Structured Clinical Interview for DSM-V-TR Axis I Disorders (SCID) [33].

All subjects had a physical and psychiatric examination. Urinalysis was done on the day of the scan to exclude the use of illicit drugs. Exclusion criteria for all participants included: female sex; history of head trauma or injury with loss of consciousness longer than one hour; learning disabilities or lack of English fluency; current or lifetime history of alcohol dependence; current or lifetime history of substance abuse or dependence; use of any recreational substances within the last month; current use or within the last month of psychotropic medication (other than an antipsychotic) with possible effects on the GABA system (such as antiepileptic drugs, benzodiazepines and antidepressants) [34] and contraindications to MRI or PET. Patients were excluded if treated with clozapine, diagnosed with an organic psychosis or had a history of neurological or psychiatric disorders other than schizophrenia, as assessed by the SCID [33]. Exclusion criteria for the antipsychotic-free patients included current or recent use of antipsychotics within the last 4 weeks or five half-lives of the drug (whichever was longer). The antipsychotic-free status was confirmed by reviewing clinical notes and pharmacy records. Healthy controls were administered the Psychosis Screening Questionnaire (PSQ) [35] and were excluded if they reported any psychotic symptom, history of psychotic illness or family history of psychosis. None of the healthy controls recruited to the study had a positive score in any of the PSQ questions.

Four patients recruited into the study were excluded from participation due to PET contraindications (*N* = 3) and commencement of clozapine treatment (*N* = 1). Therefore, the final analyses include 21 patients on antipsychotic mediation (medicated group) and 19 matched healthy controls and 10 antipsychotic-free patients (antipsychotic-free group) and 10 matched healthy controls.

### Clinical assessment

Antipsychotic doses were converted into chlorpromazine equivalent dose as previously described [36]. Symptoms were evaluated using the Positive and Negative Syndrome Scale (PANSS) [37]. Duration of untreated psychosis was quantified as the interval between first psychotic symptoms and first contact with psychiatric services [38].

### Neuroimaging evaluation

#### Image acquisition

Participants were instructed to refrain from alcohol for at least 12 h before scanning. Dynamic PET scans were acquired over 90 min using a Siemens ECAT EXACT HR (CTI/Siemens, model 962; Knoxville, TN, USA) scanner, with 63 *trans*-axial image planes covering an axial field of view of 15.5 cm. PET emission data were corrected for attenuation and scatter using a 10-min transmission scan and reconstructed using Fourier rebinning and 2D filtered back projection with a 2.0 mm kernel Ramp filter, into 23 dynamic frames (4 × 15, 4 × 60, 2 × 150, 10 × 300, and 3 × 600 s). The final reconstructed volume had voxel dimensions of 2.059 × 2.059 × 2.000 mm^3^.

During the PET acquisition, arterial blood data were sampled via the radial artery using a combined automatic-manual approach. A continuous sampling system (ABSS Allogg, Mariefred, Sweden) was employed to measure whole blood activity for first 15 min of each scan at the rate of one sample per second. Discrete blood samples were manually withdrawn at 2, 5, 10, 15, 20, 25, 30, 40, 50, 60, 70, 80, and 90 min, centrifuged and used to determine the plasma over blood activity ratio (POB). Samples at 2, 5, and 10 min were used to calibrate the two sampling modalities. Samples taken at 2, 5, 10, 20, 30, 50, 70, and 90 min were also analyzed using HPLC to calculate the plasma fraction of tracer free of metabolites (PPf). Both POB and PPf were fitted with an extended Hill model [39], while whole blood time–activity curves (TACs) were fitted using a multiexponential model [40] using the Multiblood software [41]. For each scan, a time delay was fitted and applied to the input functions (both parent and whole blood TACs) to account for the temporal delay between blood sample measurement and the target tissue data.

High resolution T1 weighted volumes were acquired using a 3T MR scanner (Magnetom Trio Syngo MR B13 Siemens 3T; Siemens AG, Germany) and a magnetization prepared rapid gradient echo sequence (TR = 2300 ms, TE = 2.98 ms, TI = 900 ms, flip angle = 9°, field of view = 256 mm, image matrix = 240 × 256) with a resolution of 1 mm isotropic. For the volume, 160 abutting straight sagittal slices were collected in an interleaved right to the left manner, resulting in whole head coverage.

### [^11^C]Ro15-4513 PET data processing

Dynamic PET data were corrected for interframe motion and aligned with the individual’s structural T1 MR image by minimizing a mutual information cost function (SPM8, Wellcome Trust Centre for Neuroimaging, http://www.fil.ion.ucl.ac.uk/spm). A neuroanatomical atlas was also co-registered to each subject’s image space (MIAKAT, http://www.imanova.co.uk) and applied to the dynamic PET data to derive regional TACs. The hippocampus was selected as our a priori region of interest since it has been implicated in the pathogenesis of schizophrenia and has a high expression of α5-GABA-A receptors relative to the rest of the brain, representing ~25% of GABA-A receptors in this region [36]. Moreover, the quantification of the α5 subunit using [^11^C]Ro15-4513 has been shown to be particularly reliable in the hippocampus (intraclass correlation coefficients (ICC) ~0.9) [42]. GABA-A receptor availability was indexed using the distribution volume (*V*_T_) of [^11^C]Ro15-4513. In agreement with previous publications [43, 44], the *V*_T_ was calculated using with the metabolite-free arterial plasma input function and the two-tissue compartmental model (2TCM) solved with a nonlinear least squares approach for the regional level and the Logan graphical method at the voxel level [45].

### Statistical analysis

Statistical analyses were performed with SPSS version 20 (IBM, Armonk, NY) for MAC OS X. The data distribution of all variables was tested using Kolmogorov–Smirnov tests and Grubbs test was used to determine if there were outlying data. Since all data were normally distributed, parametric tests were used. To test our primary hypothesis that GABA-A receptor availability would be altered in patients, an independent samples *t*-test was used to investigate if there were group differences in the *V*_T_ of [^11^C]Ro15-4513 in the hippocampus in (1) medicated patients relative to age-matched healthy controls and (2) antipsychotic-free patients relative to age-matched healthy volunteers. Independent samples *t*-tests were also used to investigate group differences in sociodemographic and clinical data. There was an outlier in the medicated patient group with high *V*_T_ values in the hippocampus (Grubbs test *p* < 0.05) and these data were excluded from further analysis. To determine whether there was an effect of group on *V*_T_ values in our exploratory analyses of other brain regions, we performed a two-way ANOVA with *V*_T_ as the dependent variable and group (patient or control) and brain region as the independent variables. Exploratory analyses were also used to investigate the association between [^11^C]Ro15-4513 and clinical data using Pearson’s product moment correlation. All data are presented as mean ± SD, and the *α* level was set for all comparisons at *p* < 0.05, corrected.

## Results

### Demographic and clinical characteristics of study participants

Four groups of participants were included in the study: twenty-one patients taking antipsychotic mediation (medicated group) and nineteen matched healthy controls and ten antipsychotic-free patients (antipsychotic-free group) and ten matched healthy controls. In the medicated group, 19 patients had a diagnosis of schizophrenia and 2 patients had a diagnosis of schizoaffective disorder. No patient had any comorbid psychiatric diagnosis as assessed by the SCID. Only one patient was taking a first-generation antipsychotic, while the remaining 20 patients were taking a second-generation antipsychotic drug (Supplementary Data, Table S1). All of the patients in the antipsychotic-free group had a diagnosis of schizophrenia and were antipsychotic-free for a period of at least 4 weeks or five half-lives of the drug (whichever was longer).

The demographic and clinical characteristics of the subjects are presented in Table 1. There were no group differences in any sociodemographic variables, or injected dose, injected mass or specific activity in medicated and antipsychotic-free patients and their respective healthy control groups.

**Table 1:** Demographic and clinical characteristics of the medicated and antipsychotic-free schizophrenia patients and their healthy comparison subjects.

	Medicated patients (*n* = 21)		Healthy controls (*n* = 19)		Test statistic	Antipsychotic-free patients (*n* = 10)		Healthy controls (*n* = 10)		Test statistic
	Mean	SD	Mean	SD	*p*	Mean	SD	Mean	SD	*p*
Age at scan (mean years, SD)	37.2	10.95	42.2	7.27	0.102	24.1	3.17	26.3	1.49	0.06
Handedness: right-handed (%)	100%		100%		1	100%		100%		1
PANSS total score	65.6	14.35	n/a	n/a	n/a	56.5	16.3	n/a	n/a	n/a
Duration of illness (months)	110.4	84.1	n/a	n/a	n/a	32.1	33.6	n/a	n/a	n/a
Medication dosage (CPZ equivalents)	328.1	160.1	n/a	n/a	n/a	n/a	n/a	n/a	n/a	n/a
Radioactivity dose (MBq)	353.4	79.4	365.8	59.0	0.58	334.9	67.3	319.3	80.3	0.64
Specific activity (GBq/μmol)	36.1	12.5	42.0	17.0	0.21	36.5	13.3	45.1	17.0	0.22
Injected mass (μg)	3.69	1.9	3.38	1.69	0.58	2.80	1.97	3.18	1.78	0.45
Total hippocampal volume (mm^3^)	10380	1012	10952	1245	0.18	10708	1270	10925	1191	0.69
Left hippocampal volume (mm^3^)	4040	400	4238	477	0.16	3952	1344	4361	371	0.36
Right hippocampal volume (mm^3^)	3839	492	3935	420	0.51	3672	1257	4270	599	0.19

*SD* standard deviation, *CPZ* chlorpromazine, *n.a*. not applicable.

### Hippocampus α5-GABA_A_Rs availability in patients and controls

**Fig. 1:**
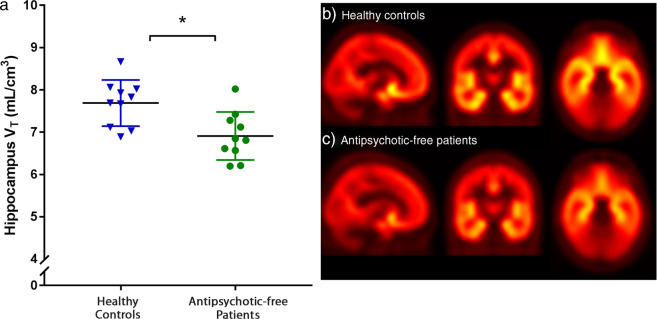
Volume of distribution (VT) values of [11C]Ro15-4513 in the hippocampus by group for antipsychotic-free patients (with mean ± SD). There was significantly lower [11C]Ro15-4513 volume of distribution (VT) in the hippocampus in antipsychotic-free patients with schizophrenia relative to healthy controls (*p < 0.05; effect size = 1.4). Group average VT parametric maps showing sagittal, coronal, and transverse images (from left to right) for: **b** healthy controls and **c** antipsychotic-free schizophrenia patients.

**Fig. 2:**
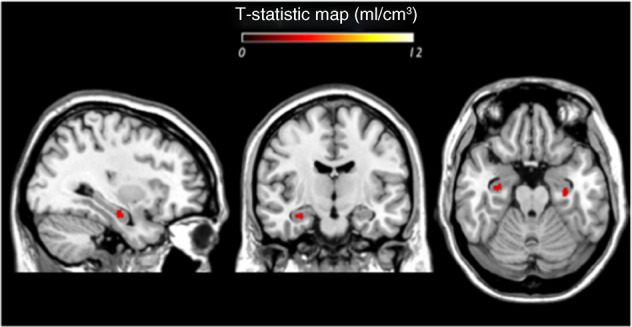
Voxelwise T-static maps showing regions within the hippocampus where *V*_T_ is significantly lower in antipsychotic-free patients relative to controls (threshold *p* = 0.05 uncorrected for illustration purposes). Images are in MNI space (voxel size: 2 mm × 2 mm × 2 mm). The hippocampal mask included 1573 voxels.

There was significantly lower [^11^C]Ro15-4513 *V*_T_ in the hippocampus of antipsychotic-free patients when compared with healthy controls (mean ± SD: 6.90 ± 0.56 vs. 7.68 ± 0.54, respectively, *p* = 0.006, effect size = 1.4; Figs. 1 and 2).

**Fig. 3:**
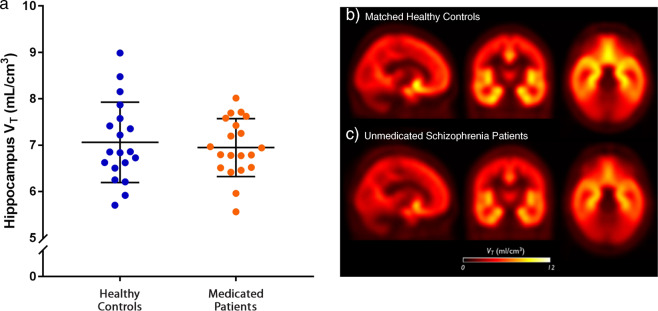
Volume of distribution (*V*_T_) values of [^11^C]Ro15-4513 in the hippocampus in medicated patients and healthy controls. **a** Scatter plots showing the volume of distribution (*V*_T_) values of [^11^C]Ro15-4513 in the hippocampus by group for medicated patients (with mean ± SD). There were no significant differences between medicated patients with schizophrenia and healthy controls. Group average *V*_T_ parametric maps showing sagittal, coronal, and transverse images (from left to right) for: **b** healthy controls and **c** antipsychotic-free schizophrenia patients.

In contrast there was no significant group difference in the [^11^C]Ro15-4513 *V*_T_ between the medicated patients and healthy controls (mean ± SD: 6.94 ± 0.62 vs. 7.06 ± 0.86, *p* = 0.64) (Fig. 3). There was also no significant difference in α5-GABA_A_Rs availability between the medicated and antipsychotic-free schizophrenia groups (*p* = 0.54).

### Correlations between hippocampus α5-GABA_A_Rs availability and clinical characteristics

**Fig. 4:**
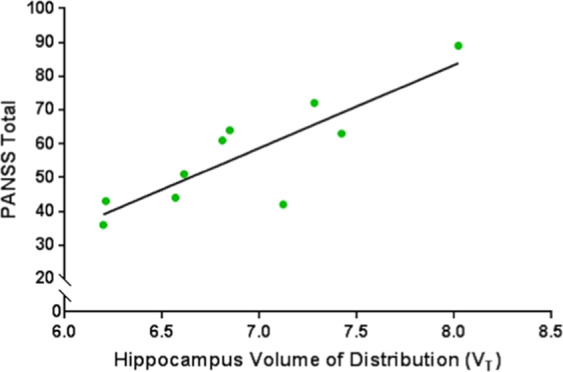
Correlation between volume of distribution (*V*_T_) values of [^11^C]Ro15-4513 in the hippocampus and total PANSS symptom severity scores in antipsychotic-free patients with schizophrenia. There was a significant positive correlation between hippocampal *V*_T_ and symptom severity (*r* = 0.72; *p* < 0.05).

We found no significant correlation between [^11^C]Ro15-4513 *V*_T_ and the exposure to antipsychotics, as measured in chlorpromazine equivalents doses (*r* = 0.32; *p* = 0.22). There was also no significant correlation with *V*_T_ and duration of illness in either the antipsychotic-free (*r* = 0.105; *p* = 0.78) or the medicated (*r* = −0.02; *p* = 0.91) groups. In the antipsychotic-free patient group, there was a significant positive correlation between [^11^C]Ro15-4513 *V*_T_ and severity of symptoms, as assessed by the total PANSS score (*r* = 0.72; *p* = 0.044) (Fig. 4), which was not significant in the medicated patient group (*r* = 0.22; *p* = 0.38). There was also no significant correlation between [^11^C]Ro15-4513 *V*_T_ and age in either the medicated (*r* = −0.20; *p* = 0.19) or the antipsychotic-free (*r* = 0.16; *p* = 0.49) groups.

### Hippocampus volume

There were no significant differences in hippocampal volumes between antipsychotic-free patients relative to healthy controls and between medicated patients and healthy controls (Table 1). There were also no significant correlations between hippocampal volumes and the [^11^C]Ro15-4513 *V*_T_ in the hippocampus in either antipsychotic-free patients (*r* = −0.035; *p* = 0.92) or medicated patients (*r* = 1.99; *p* = 0.38)

### Other brain regions

In antipsychotic-free patients relative to healthy controls, there was a significant main effect of group on *V*_T_ measures across all ROIs (*F* (1, 144) = 5.73, *p* = 0.01). There was also a significant main effect of region on *V*_T_ measures across all ROIs (*F* (7, 144) = 179, *p* < 0.001). However, the group × region interaction was not significant (*F* (7, 144) = 0.61, *p* = 0.74), and there were no significant differences in the posthoc pair-wise comparisons for individual regions (Supplementary Data, Table S2). In the medicated patient group, there was no significant effect of group on *V*_T_ measures across all ROIs (*F* (1, 304) = 0.50, *p* = 0.47). However, there was a significant main effect of region on *V*_T_ across the brain (*F* (7, 304) = 161, *p* < 0.001) but the group × region interaction was not significant (*F* (7, 304) = 0.94, *p* = 0.47), and there were no significant differences in the posthoc pair-wise comparisons for individual regions (Supplementary Data, Table S2).

## Discussion

Our results show that GABA-A receptor availability, measured with a tracer selective for the α5-GABA-A receptor subunit, is significantly lower in the hippocampi of antipsychotic-free schizophrenia patients, and directly correlated with symptom severity. However, α5-GABA_A_Rs availability is not significantly altered in antipsychotic-treated patients when compared with controls.

Our results extend postmortem findings showing lower mRNA and protein levels of α5-GABA_A_Rs in schizophrenia patients [23, 24] to show evidence for lower hippocampal uptake of an α5-GABA_A_Rs selective tracer in vivo in untreated patients with schizophrenia. However, our results are not consistent with the only previous [^11^C]Ro15-4513 PET study in schizophrenia patients [28], where no differences in [^11^C]Ro15-4513 BP were observed between antipsychotic-free patients and healthy controls. The lack of group effects in this previous study may be due to the use of simplified reference tissue model (SRTM) with the pons as the reference tissue to quantify [^11^C]Ro15-4513 BP. This approach relies on a number of assumptions which can easily lead to biased estimates if violated [46]. Moreover, the ICC across brain regions range between 0.35 and 0.80 when using SRTM, which indicates lower reliability than the approach we used, where ICC values are >0.82 [42, 47]. The use of the pons as a reference region for [^11^C]Ro15-4513 PET has also been subject to criticism, since it may lead to the underestimation of target binding [19, 46, 47]. For these reasons, the SRTM approach used in the Asai et al. study may have lacked sensitivity to detect group differences.

Interestingly, our study showed no significant differences in [^11^C]Ro15-4513 *V*_T_ in medicated schizophrenia patients when compared with controls. Taken with our findings in the antipsychotic-free patients and other findings of lower GABA-A receptor prior to treatment [48], these results could suggest that there has been a normalization of α5-GABA_A_Rs levels with antipsychotic treatment. In addition, reduced GABA release, as assessed using a tiagabine challenge, was seen in antipsychotic-naive individuals but not in antipsychotic-treated patients [15]. Previous literature has also shown evidence of altered GABA levels using 1H-MRS in unmedicated patients but not in medicated patients [32]. Moreover, 4 weeks of exposure to antipsychotic treatment has also been found to normalize GABA levels in patients [32]. Post-mortem studies also find differences between treated and untreated patients with schizophrenia, showing a positive correlation between the density of GAD and antipsychotic dose and higher expression levels of GAD67 and GABA-A protein subunits in antipsychotic-treated patients relative to unmedicated patients [49]. However, it is important to note that both in our study and in these prior studies, the antipsychotic-treated group had a longer duration of illness than the untreated group. As such, it is not possible to disentangle duration of illness from treatment effects. However, in our study, we found no significant associations between α5-GABA_A_Rs availability and duration of illness, indicating that antipsychotic treatment could contribute to the lack of group differences in the treated patients. This is supported by preclinical findings showing an increased expression of GAD67 and increased GABA-A receptor binding in rats exposed to a range of different antipsychotics [50] and by clinical studies showing that exposure to 4 weeks of antipsychotic treatment normalizes GABA levels in schizophrenia patients [32]. However, longitudinal studies in patients are needed to test the hypothesis that there are changes in GABA-A receptor levels over time and if they are associated to exposure to antipsychotic medication.

### Strengths and limitations

A strength of the study was that we included a large sample for a PET study and obtained arterial input functions [19]. In our study, we used the 2TCM model, which has been shown to have good reliability for [^11^C]Ro15-4513 quantification (ICC ranging from 0.83 to 0.88), and *V*_T_ values derived from this model show a close, linear relationship with α5-GABA_A_Rs specific binding [19]. One potential limitation on generalizability is that we only included males, as there are sex differences in GABA-A receptor expression [30]. Future studies are needed to test if our findings generalize to female patients as well. Although both groups are matched for age, there is a trend level difference in age between antipsychotic-free patients and healthy controls (*p* = 0.06). Importantly, the *V*_T_ values in our healthy control cohorts are similar to the ones reported in other studies using the [11C]Ro15-4513 PET tracer [51, 43], indicating that our findings are not driven by abnormal values in our controls. Furthermore, α5-GABAARs show developmental reductions and, supporting this, in healthy volunteers there is an inverse relationship between age and [^11^C]Ro15-4513 *V*_T_ values. The lack of group differences in the chronic patients taken with the lower *V*_T_ values could suggest disruption of the normal age-related reductions in α5-GABAARs. The antipsychotic-free period in the antipsychotic-free group was 4 weeks or over five half-lives (whichever was longer), and no antipsychotic taken in the medicated group has significant affinity for GABA-A receptors (all Ki values > 10,000) [52–55], indicating that direct effects of antipsychotic drugs on GABA-A receptors are unlikely to explain our findings. We did not find any significant difference in hippocampal volume, suggesting that partial volume effects are unlikely to account for our group differences. Furthermore, although a reduction in hippocampus volume in schizophrenia is commonly reported [56], in our study there were no significant volumetric differences in hippocampal volume between patients and controls. This can be explained by the hippocampus being one of the brain areas with greater structural variability [57]. Finally, [^11^C]Ro15-4513 is a highly selective α5-GABA_A_Rs PET radiotracer, with α5 representing around 60–70% of the specific binding in α5 rich regions [19], and its affinity for this subunit is ~10- to 20-fold greater than to other subtypes of the GABA-A receptor [16, 17]. However, it also demonstrates measurable in vivo binding for the α1 to α4 and α6 receptor subtypes [16, 17]. Overall, although the [^11^C]Ro15-4513 *V*_T_ reflects the net effect of the availability of these different GABA-A receptors, the 20-fold greater affinity of [^11^C]Ro15-4513 for α5-GABA_A_Rs and the enriched expression of α5-GABA_A_Rs in the hippocampus indicates the lower [^11^C]Ro15-4513 hippocampal availability is likely to predominantly reflect lower α5-GABA_A_R levels.

### The interpretation of the findings and their implications for understanding schizophrenia

GABAergic neurotransmission in the hippocampus plays a fundamental role in inhibiting the neuronal activity of pyramidal glutamatergic cells, through the activation of postsynaptic GABA receptors [58, 59]. The hippocampus has a particularly high concentration of α5-GABA_A_Rs relative to other brain regions, constituting up to 25% of all GABA-A receptors in the hippocampus. Although other receptor subtypes are also present [60], α5 represents around 60–70% of the specific binding of [^11^C]Ro15-4513 in the hippocampus [19, 61]. While α1 and α2-GABA_A_Rs are located synaptically and involved in the phasic inhibition of pyramidal cells, α5-GABA_A_Rs are located mainly at extrasynaptic sites [19, 62]. At these sites, α5-GABA_A_Rs are predominantly involved in generating a tonic inhibitory current [63–65] to regulate the excitability of pyramidal neurons [66, 67] and decrease network excitability. Interestingly, this tonic inhibition has been shown to be at least equally important in the regulation of pyramidal cells firing as phasic inhibition [63]. α5-GABA_A_Rs deficient mice show reduced tonic inhibition resulting in hippocampus hyperexcitability, which can be reversed by pharmacologically increasing the tonic current [67]. The lower α5-GABA_A_ receptor levels we observe may therefore contribute to reductions in tonic inhibition, leading to an increase in hippocampal hyperexcitability. This interpretation is supported by the previous literature implicating the hippocampus in the pathophysiology of schizophrenia including changes in hippocampal volume [68] as well as changes in regional cerebral blood flow [69] and blood oxygen level-dependent signal [70]. Taken together these suggest that lower α5-GABA_A_Rs levels could lead to a state of excessive excitation [59, 62]. Our findings are therefore consistent with hypotheses that a reduction in GABAergic neurotransmission disinhibits glutamatergic pyramidal neurons, resulting in the loss of synchronous cortical activity, and subcortical dopamine dysregulation. Furthermore, the activation of α5-GABA_A_Rs by a PAM has been shown to reverse the dopaminergic and behavioral abnormalities observed in the MAM model [25–27]. These findings highlight the therapeutic potential of targeting GABA function in schizophrenia [1, 59]. We found a significant positive correlation between α5-GABA_A_Rs availability and symptom severity in antipsychotic-free patients. Whilst this supports a link between α5-GABA_A_Rs dysregulation and the clinical expression of the disorder, the direction of the association is not consistent with a simple model of α5-GABA_A_Rs hypofunction leading to hippocampal overactivity and symptoms. It was recently shown, using a viral-mediated gene transfer, that an increase in the expression of α5-GABA_A_Rs normalizes VTA dopamine cell population activity [71]. Thus, one explanation that could account for both the lower [11C]Ro15-4513 binding in patients and a positive correlation with symptoms is that low α5-GABA_A_R levels are a vulnerability factor but that once the disorder develops there is a compensatory increase in α5-GABA_A_R levels in response to dysregulation of other systems. This is consistent with the role of α5-GABA_A_Rs in providing tonic regulation of pyramidal neurons [72]. A longitudinal study is needed to test this model further. Finally, our exploratory analysis in other brain regions also showed a significant difference between antipsychotic-free patients and controls in the omnibus test, suggesting there may be GABA-A receptor alterations in other brain regions in schizophrenia. However, no individual region was significantly different in the pair-wise comparisons, which could reflect the fact that the expression of α5-GABA_A_Rs is lower in these regions [33].

### Conclusions and future directions

This study provides evidence that antipsychotic-free patients with schizophrenia have lower GABA-A receptors in the hippocampus, predominantly of the alpha-5 subtype, that is not seen in antipsychotic-treated patients. These findings are consistent with the hypothesis that GABA hypofunction underlies the pathophysiology of the disorder, and highlights the potential of GABAergic modulators as therapeutic targets for schizophrenia [72].

## Supplementary information

Supplementary Table S1

Supplementary Table S2
