# Supplementary material for: GABA-A receptor differences in schizophrenia: a positron emission tomography study using [11C]Ro154513
Source: Mol Psychiatry. 2020 Apr 15;26(6):2616–25. doi: 10.1038/s41380-020-0711-y (PMC8440185; doi:10.1038/s41380-020-0711-y)
Supplement: Supplementary file 1 — Supplementary Table S1 [file 41380_2020_711_MOESM1_ESM.docx]

|  | | |
| --- | --- | --- |
|  | **Medicated Patients (n= 21)** | |
| Type of Antipsychotic | N | % |
| Olanzapine | 6 | 28% |
| Risperidone | 5 | 24% |
| Paliperidone | 4 | 19% |
| Aripiprazole | 3 | 14% |
| Amisulpride | 2 | 10% |
| Flupenthixol | 1 | 5% |
|  | | |

*Table S.1. Type and percentage of antipsychotic use by medicated patients with schizophrenia*
