# Supplementary material for: GABA-A receptor differences in schizophrenia: a positron emission tomography study using [11C]Ro154513
Source: Mol Psychiatry. 2020 Apr 15;26(6):2616–25. doi: 10.1038/s41380-020-0711-y (PMC8440185; doi:10.1038/s41380-020-0711-y)
Supplement: Supplementary file 2 — Supplementary Table S2 [file 41380_2020_711_MOESM2_ESM.docx]

|  | | | | | | | | | | |
| --- | --- | --- | --- | --- | --- | --- | --- | --- | --- | --- |
|  | Medicated Patients  (n= 21) | | Healthy Controls  (n= 19) | | Test statistic | Antipsychotic-free Patients  (n= 10) | | Healthy Controls  (n= 10) | | Test statistic |
| ROI | Mean | SD | Mean | SD | p | Mean | SD | Mean | SD | p |
| Amygdala | 6.67 | .56 | 7.10 | .85 | .063 | 6.55 | .68 | 6.90 | .65 | .25 |
| Anterior Cingulate cortex | 7.83 | 1.46 | 7.52 | 1.10 | .45 | 7.80 | .64 | 8.06 | .51 | .34 |
| Caudate nucleus | 3.73 | 0.59 | 3.63 | 0.99 | .68 | 3.41 | .34 | 3.99 | .37 | .12 |
| Frontal Lobe | 5.33 | 0.64 | 5.10 | 0.50 | .22 | 5.41 | .33 | 5.32 | .40 | .58 |
| Occipital Lobe | 4.42 | 0.40 | 4.35 | 0.45 | .61 | 4.49 | .32 | 4.54 | .28 | .71 |
| Putamen | 5.35 | 0.88 | 5.49 | 0.94 | .61 | 5.02 | .62 | 5.05 | .59 | .92 |
| Temporal Lobe | 6.37 | 0.60 | 6.21 | 0.76 | .47 | 6.41 | .45 | 6.55 | .35 | .46 |
| Thalamus | 3.18 | 0.64 | 2.97 | 0.35 | .21 | 3.18 | .28 | 3.31 | .25 | .31 |
| SD = Standard Deviation | | | | | | | | | | |

*Table S.2. [^11^C]Ro15-4513 volume of distribution (V_T_) values in medicated and antipsychotic-free schizophrenia patients and healthy comparison subjects across multiple brain regions*
